# Designing Virtual, Moderated Studies of Early Childhood Development

**DOI:** 10.3389/fpsyg.2021.740290

**Published:** 2021-10-11

**Authors:** Liesbeth Gijbels, Ruofan Cai, Patrick M. Donnelly, Patricia K. Kuhl

**Affiliations:** ^1^Department of Speech & Hearing Sciences, University of Washington, Seattle, WA, United States; ^2^Institute for Learning & Brain Sciences, University of Washington, Seattle, WA, United States

**Keywords:** virtual, moderated, development, early childhood, online

## Abstract

With increased public access to the Internet and digital tools, web-based research has gained prevalence over the past decades. However, digital adaptations for developmental research involving children have received relatively little attention. In 2020, as the COVID-19 pandemic led to reduced social contact, causing many developmental university research laboratories to close, the scientific community began to investigate online research methods that would allow continued work. Limited resources and documentation of factors that are essential for developmental research (e.g., caregiver involvement, informed assent, controlling environmental distractions at home for children) make the transition from in-person to online research especially difficult for developmental scientists. Recognizing this, we aim to contribute to the field by describing three separate moderated virtual behavioral assessments in children ranging from 4 to 13years of age that were highly successful. The three studies encompass speech production, speech perception, and reading fluency. However varied the domains we chose, the different age groups targeted by each study and different methodological approaches, the success of our virtual adaptations shared certain commonalities with regard to how to achieve informed consent, how to plan parental involvement, how to design studies that attract and hold children’s attention and valid data collection procedures. Our combined work suggests principles for future facilitation of online developmental work. Considerations derived from these studies can serve as documented points of departure that inform and encourage additional virtual adaptations in this field.

## Introduction

Over the past decades, technological advancements have expanded the scale and scope of academic research. A body of literature between 1995 and 2005 proposed a series of benefits and disadvantages associated with the initial wave of Internet-based research (Hewson et al., 1996; Reips, 2001, 2002; Duffy, 2002; Kraut et al., 2004), which underscored a time when digital research was relatively novel and small-scale. Despite the growing popularity of much online work following the rise of digital media in the 21st century, research in the field of child development stayed relatively resistant, and digital formats of developmental research have only recently been demonstrated (Scott et al., 2017; Scott and Schulz, 2017; Sheskin and Keil, 2018; Gweon et al., 2020; Nussenbaum et al., 2020; Rhodes et al., 2020; Sheskin et al., 2020). Further, established methodological adaptations in this field are largely characterized as immature, especially in the adoption and validation of online behavioral assessments (Scott and Schulz, 2017; Nussenbaum et al., 2020; Rhodes et al., 2020).

In 2020, as the COVID-19 pandemic led to reduced social contact, causing many research laboratories to close, the scientific community began to investigate online research methods that would allow continued work. Remote, digital modalities have been recognized as viable substitutions for in-person research settings (Reips, 2001, 2002; Sheskin and Keil, 2018). In comparison with laboratory-based research methods, advantages associated with general online research (e.g., reduced operating costs, increased access to diverse populations, and reduction in experimenter effects) have been reported (Reips, 2002; Bohner et al., 2002). Accompanying the recent rising trend of remote research practice, these advantages make it possible to envision a future of advanced remote methodologies for developmental work.

However, shifting from in-person to remote modalities is not without challenges. For example, Reips (2002) identified experimental control and attrition as common concerns in online research. In particular, remote behavioral measures tend to introduce additional confounds which are often attributed to increased variability in research environment and equipment. Further, online adaptations of developmental studies require nuanced, age-specific considerations such as accounting for children’s attention span and cognitive load in the task design and administration (Gibson and Twycross, 2008).

Although solutions have been proposed to address some of the challenges (Reips, 2002), peer-reviewed methodological reports of adaptation from in-person to online developmental studies are rather limited, awaiting substantial input. Recognizing the lack of documented observations from existing virtual research and its potential to deter future implementations of online developmental work, we aim to contribute to the field by describing three researcher-moderated virtual assessments in children ranging from 4 to 13years of age, encompassing assessments of their speech processing skills and reading fluency. The varied domains, in combination with the age groups targeted by each study, required different methodological approaches. However, the success of our remote adaptations shared certain commonalities regarding informed consent, study designs that attract and hold children’s attention, and valid data collection procedures. Through this work, we hope to suggest principles for future facilitation of online developmental research, and we believe that considerations derived from these three studies can serve as documented points of departure that inform and encourage additional virtual adaptations in this field.

The three studies included in this paper sought to adapt their original in-person task designs for remote facilitation with researcher moderation. While the moderated format was appropriate for these studies, both moderated and unmoderated designs have their pros and cons, and we encourage developmental scientists to make decisions with regard to the degree of moderation while facilitating online child studies. Compared to moderated studies, unmoderated or fully automated studies are less work-intensive during the research appointments, but it may require more preparation work in task automation and involve additional steps of data processing. Elimination (or lessening) of researcher involvement is advantageous in bias removal, as it is often replaced by consistent machine-delivered instructions. This facilitates the comparison across replications of unmoderated studies (Rhodes et al., 2020). However, for the same reason that makes unmoderated formats appealing to some, the lack of researcher real-time involvement also presents several challenges.

### Informed Consent and Data Security

Ethics of non-therapeutic research involving children are a delicate issue, as children are vulnerable and would likely not benefit directly from participation (Lambert and Glacken, 2011). In language suitable for the intended individual, informed consent/assent should communicate the study’s purpose and procedures, associated benefits and risks, confidentiality, safety, etc. Additionally, when appropriate, the researcher or caregiver may need to verbally communicate the informed consent, which is often crucial to ensuring participants’ understanding, as the informed consent ought to be viewed as a process rather than a product, beyond signature collection (Whitehead, 2007; Gibson and Twycross, 2008).

For many virtual studies, using online applications, such as REDCap, are an appealing way to collect e-consent and to build and manage online databases. A lot of web tools come with built-in privacy measures, allowing digital consent to be completed efficiently and stored securely. On platforms such as Pavlovia and Gorilla, documentation of major identifying information can stay detached from research data, and it is often possible to record the consent process and data collection separately (Sheskin and Keil, 2018). However, it is generally difficult for unmoderated consent processes to create space for researchers to interact with participants and address participants’ questions or concerns. In addition, experimental processes that rely on human-machine interactions (e.g., text-based or video/audio recording) alone could run a higher risk of technical error, resulting in corrupted recordings, for example. In contrast, a moderated process enables candid researcher-participant communication and provides flexibility for procedural adjustments (guided by a well-designed rubric), which is frequently needed due to increased variability and unpredictability of virtual studies in home environments.

Protecting participants’ privacy and data confidentiality is among the top priorities in human subject research. Remote consent processes in recent years have shown varying formats. Some researchers opt for digital acquisition of text-based consent *via* email (Nussenbaum et al., 2020) or online secure databases (Donnelly et al., 2020a, 2020b), and others acquire verbal consent and assent using automated video and audio recording (Scott and Schulz, 2017; Rhodes et al., 2020). While research moderation is not required for either option, the latter, when unmoderated, is subjected to technical issues with video/audio recording, potentially resulting in invalid data if not detected promptly (Rhodes et al., 2020). Scott and Schulz (2017) reported that up to 16% of their data were discarded due to inadequate consent recordings. In contrast, in addition to audio or video recording documentations (Sheskin and Keil, 2018), researcher observation and natural dialogues during moderated consent procedures help the researcher detect and address technical issues and ensure understanding of informed consent.

In addition, experimental stimuli and research data that are delivered and collected digitally are subjected to additional ethical scrutiny, specifically regarding data security. Some study designs may require transportation of research equipment or digital transfer of data files. In these cases, encrypting the devices and data files (e.g., using passwords or proprietary software) can significantly lower security risks, and related considerations are growing in prominence as new technologies increasingly deliver utility in research methods. As our capabilities are being enhanced rapidly, the scientific community needs to continually assess the implications of technologically enabled advancements in human subject research.

### Experimental Control and Parental Involvement

Additionally, experimental control concerns are presented in traditional research settings and highlighted even more in virtual environments. For example, whereas it is fairly straightforward to manipulate the acoustic environment in a laboratory’s sound booth, it is impossible to obtain the same level of control in participants’ homes. A realistic attempt would be to instruct caregivers to prepare a “quiet room” for the research appointment. In addition to audible noises, families may have different levels of visual and tactile distractions at home (e.g., siblings or pets). Furthermore, unless experimental equipment is specified or provided for the participants, technical device differences (e.g., headphones, Internet connection stability, screen sizes) also need to be considered.

### Motivation and Sustained Attention

Probably one of the main reasons for the slow move to online research in developmental work is that experimental designs involving children are typically more complex than those involving adults. A major challenge for child development researchers is how to best engage participants, remove distractions, and motivate participation given age-specific attention spans.

Interactions between the participant and researcher may be helpful in maintaining the child’s interest level. Developmental research studies, especially ones targeting auditory or visual perception, can benefit from researcher observation even if the task itself is fully automated. In a moderated session, the researcher-observer would be able to note any circumstances or issues that might come up and adjust as needed, whether it be troubleshooting technical difficulties, regulating caregiver involvement, clarifying task instructions, or introducing necessary breaks.

Adapting developmental research for online environments inevitably introduces tangible changes to a study’s experimental design and setup, but perhaps equally important is its impact on a socio-psychological aspect of human subject research, the researcher-participant relationship. Traditionally in a laboratory environment, face-to-face interactions can often motivate participation. While social interactions through a screen are often perceived as “flattened” and cannot fully replace their in-person counterparts, it is still possible to enhance researcher-participant relationships and to foster participant engagement and motivation through researcher moderation of remote studies. Notably, in studies involving children who struggle with unfamiliar surroundings (e.g., children with autism), the introduction of a stranger (i.e., the researcher) and a new environment (i.e., the laboratory) can be intimidating at times and interfere with the validity in data collection. In these cases, virtual assessment is an especially advantageous alternative, as it allows for in-home research participation, and can reduce or remove the perception of stranger interaction (Rhodes et al., 2020).

### Validity of Online Adaptations

Given the variety of developmental behavioral work and the limited resources for online adaptations available, questions arise regarding the validity of these adaptations. Several attempts have been made to compare in-person and remote work (Sheskin and Keil, 2018; Rhodes et al., 2020; Yeatman et al., 2021), which highlighted some important questions. Considerations for task design, stimulus presentation, attention maintenance, and results interpretation are all crucial to ensure a study’s validity. A good example that warrants caution is the interpretation of norm-referenced tasks when assessed remotely. Examples of these are intelligence tests, reading assessments, vocabulary assessments, etc. Although big companies like Pearson assessments have started to offer some tasks remotely with written guidelines, they warrant against interpretation of the norms:

A spectrum of options is available for administering this assessment *via* telepractice; however, it is important to consider the fact that the normative data were collected *via* face-to-face assessment. Telepractice is a deviation from the standardized administration, and the methods and approaches to administering it *via* telepractice should be supported by research and practice guidelines when appropriate (Pearson, 2021).

As such, interpretation of these norms when moved online should be deliberated prior to implementation.

In this paper, three different virtual studies will be discussed. Each study was initially conceived and developed for in-person environments and subsequently moved online. The original laboratory-based research plans will be summarized, along with adaptations made to enable remote facilitation. The studies targeted different questions and distinct age groups, which led to different approaches. Although the results of these studies are very promising and will each contribute to their field independently, the focal point of this paper is the adaptations we made to the three studies (Section “Procedural Modifications for Online Studies”), our data regarding their success and validity (Section “Methods; Developing Remote-Friendly Measures for Moderated, Developmental Studies”), and our resulting perspective on future implementations of virtual studies (Section “Discussion”). Through this paper, our ultimate aim is to motivate a continuance of remote developmental research, post-pandemic.

## Procedural Modifications for Online Studies

To represent the vast array of developmental research in this paper, we selected three distinct studies that varied in research goals and participants’ demographics. An Imitation study (see Section “Assessment of Vocal Imitation of Native and Nonnative Vowels (Cai and Kuhl, in Prep.)”) focusing on speech acquisition (age 4), an Audiovisual (AV) study (see Section “Audiovisual Speech Processing in Relationship to Phonological and Vocabulary Skills Gijbels et al., in Press.)”) focusing on speech perception (age 6–7), and a Reading study (see Section“A Symbolic Annotation of Vowel Sounds for Emerging Readers (Donnelly et al., 2020b)”) focusing on bringing digital tools completely online (age 8–13) will be described. Each study’s research questions, study designs, and modifications made for their virtual implementation will be outlined in Section “Procedural Modifications for Online Studies”, specific methodological adaptations will be expanded further in Section “Methods; Developing Remote-Friendly Measures for Moderated, Developmental Studies”, and the three studies will be joined together in Section “Discussion” to draw general guiding principles for future online behavioral research.

### Assessment of Vocal Imitation of Native and Nonnative Vowels (Cai and Kuhl, in Prep.)

Vast differences have been observed in second language (L2) learners’ ability to imitate novel sounds – while the majority of learners exhibit and maintain a foreign accent throughout their lifetime, some are able to produce accurate L2 pronunciations to a near-native level. These individual differences have been previously characterized as largely innate and fixed (Abrahamsson and Hyltenstam, 2008). While a number of recent published accounts have attempted to identify, in part, correlates of this talent variability (Christiner and Reiterer, 2013; Hu et al., 2013; Franken et al., 2015; Ghazi-Saidi and Ansaldo, 2017), efforts have been somewhat scattered. And despite its prevalence to the foundational research in speech perception and production, vocal imitation remains an understudied topic.

In this study, we investigated four-year-old typically developing (TD) children’s (*N*=57) ability to imitate vowel sounds, both native and nonnative, to understand young children’s sensorimotor knowledge of speech. The intent of the study was to understand how children’s ability to imitate speech relates to age, language history, and other environmental factors. The specific aims were to: (1) measure the acoustic details of children’s imitated vowels and assess the acoustic distance between their productions and those of the model they were imitating (2) determine whether children’s abilities differed for native vs. nonnative vowels, and (3) investigate individual differences in speech imitation ability among young children.

A laboratory-based format of this study was carried out during the initial pilot phase. Upon arrival at the laboratory, parents were first asked to complete a questionnaire, which surveyed environmental factors such as socio-economic status and language background. Then, the speech imitation task involving child participants was administered *via* an animal puppet theater set up in a sound booth. To deliver the auditory and visual stimuli, the researcher operated the animal puppet’s mouth behind the puppet theater, “lip-syncing” the puppet to pre-recorded speech sounds played through the speakers. A research assistant sat beside the child facing the puppet theater and assisted the participant as needed. Two video cameras, a pair of audio speakers, and a studio-quality microphone were set up in the booth. In an observation room next door, caregivers were invited to watch the live task procedures on a TV screen. This setup allowed parents to stay informed of their children’s behaviors or needs while avoiding unnecessary interference to the study session. This procedure worked during the pilot stage of this experiment, and 4-year-old children demonstrated their ability and willingness to engage in the task.

**Figure 1:**
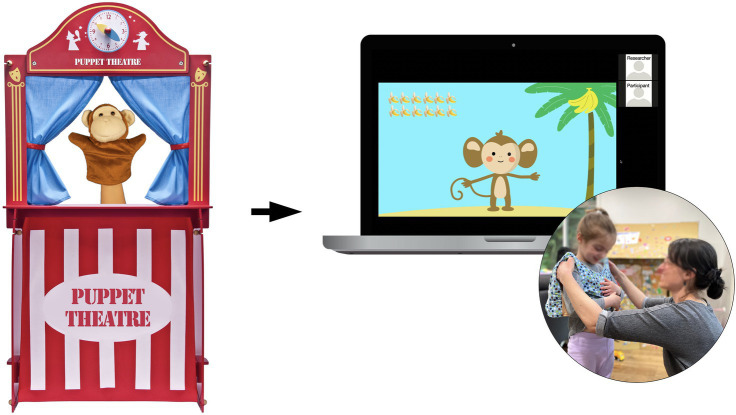
Visualization of the Imitation Study. Digital cartoon animation adapted from in-lab puppet theater setup, used to deliver auditory stimuli remotely via Zoom during the imitation task. Speech data collected via LENA vests and recorders worn by child participants (Cai and Kuhl, in prep).

In response to the public health crisis posed by COVID-19, the study was adapted digitally to accommodate remote testing. We modified the parental survey format, the protocol for parental involvement, and the means of video and audio recording of experimental sessions. Parental questionnaires were conducted digitally using a secure online portal, and the speech imitation task took place over Zoom. In the modified, online version of the imitation task, instead of plush puppets, participants interacted with animal cartoon characters on the researcher’s computer screen (*via* screen share), repeating vowel sounds after them, some “native,” and some “nonnative” to the child’s language (see Figure 1). In speech perception and production experiments, developing reliable audio systems is central to achieving consistent stimulus presentation and quality data acquisition. The key measurement in this study is the acoustic distance between the vowel target (i.e., model) and imitation (i.e., production), which is calculated using formant frequency values of the vowel target and of the imitation. Recognizing the variability in hardware and software configurations across participants, in addition to using the video and audio recording system built into the Zoom video conferencing platform, we also mailed individual pocket-sized audio recorders – the Language ENvironment Analysis system (LENA™, the LENA Research Foundation, Boulder, CO) – to the participating families to capture the children’s speech productions in their environment more accurately and consistently. Additionally, given the participants’ young age and the virtual administration of an interactive task, parents assisted with facilitation of the appointment when needed.

Online adaptations of the study were successfully implemented. Forty-six out of 57 participating subjects were included in the analysis, with a resulting total of over 7,000 utterances examined, and audio files retrieved from the LENA recorders provided adequate acoustic information for the purpose of vowel formant analysis (see “Validity of online adaptations”).

### Audiovisual Speech Processing in Relationship to Phonological and Vocabulary Skills

The benefits of audiovisual (AV) speech perception, more specifically, having access to the (Gijbels et al., in press) articulation movements when the auditory speech signal is degraded by noise, have been well studied in adults (see Grant and Bernstein, 2019 for a review). And although we know that infants (Kuhl and Meltzoff, 1984) and children (Lalonde and Werner, 2021 for a review) are sensitive to AV speech information, the size and the presence of an actual AV speech benefit have been debated (Jerger et al., 2009, 2014; Fort et al., 2012, Ross et al., 2011; Lalonde and McCreery, 2020). More specifically, 5-to 8-year-olds show highly variable results when completing audiovisual speech perception tasks. As suggested by Lalonde and Werner (2021), these results might be explained by extrinsic factors as task complexity, intrinsic factors (i.e., individual developmental skills) or the combination of both (i.e., general psychophysical testing performance).

**Figure 2:**
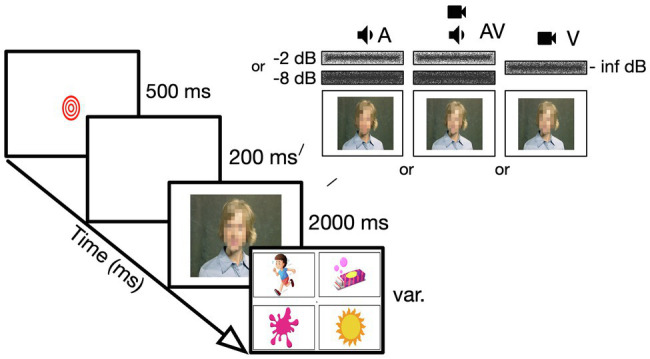
Visualization of the AV Study Experimental set up of the AV Study in both laboratory and online. Audio-only, audiovisual, or visual-only stimulus presentation of one-syllable words in speech-weighted noise, followed by a 4AFC answer screen (Gijbels et al., in press).

The specific aims of this study were to assess (1) whether TD children in first grade (*N*=37; 6–7years old) show AV speech enhancement in a noisy environment when a task is presented with low cognitive and linguistic demands (i.e., extrinsic factors) (2) whether individual variability in AV gain is related to intrinsic developmental factors (Jerger et al., 2009; Ross et al., 2011), or to (3) the combination of intrinsic and extrinsic factors. To address these questions, the participants completed an AV speech perception task (see Figure 2). In this task, audio-only (i.e., stimulus word + speech-weighted noise + still image), audiovisual (i.e., stimulus word + speech-weighted noise + matching video), or visual-only (i.e., speech-weighted noise + video) stimuli were presented in 200 trials, broken up in 10 blocks. The stimulus was followed by four answer options (i.e., one a correct answer, two options were related in word form, and a random answer option). Additionally, participants completed standardized measures of vocabulary (Expressive Vocabulary Test; EVT-3; Williams, 2019) and phonological awareness skills (Phonological and Print Awareness Scale; PPA; Williams, 2014), and a third control auditory psychophysical task, that was very similar in setup to the AV task but had no speech or visual component to it. The cognitive and linguistic demands were limited by using a closed set (four-alternative forced choice; 4AFC) picture pointing task, with a stimulus set of consonant-vowel-consonant words that are well known by typically developing children of this age (Holt et al., 2011).

In a laboratory setting, we would measure individuals’ behavioral and psychophysical performance in a quiet, controlled environment (i.e., sound booth) to ensure the reliability of stimuli and response. Conducting the tasks in a quiet room in the laboratory provides the opportunity to assess baseline control of hearing thresholds and visual acuity, eliminates potential interference (e.g., background noise), avoids unintended asynchrony of auditory and visual stimuli, and maintains exact output levels and quality of all stimuli using a calibrated computer. It also allows interpretation of normed behavioral tests, as they can be assessed according to the manual. Interference from parents would be limited as they would wait in the waiting room and instructions and assessment would be provided by a trained research assistant.

For both the in-person and the online version of the experiment, the stimulus presentation followed by 4AFC answer options would look identical. Also, the number of breaks (stimulus blocks) and catch trials were kept consistent. However, to move the tasks to a virtual environment, the tools for stimulus presentation (i.e., assessment format), data interpretation methods, and parental involvement had to be re-envisioned. Participants would complete the tasks at home, in front of their personal computer in a varied environment (i.e., background noise). Parents were instructed before and during the moderated session to provide a “controlled” and consistent environment. They would act as technical support and report presented technical hiccups, but also take over tasks that the research assistant would normally provide in the laboratory (e.g., providing mouse control when the child had insufficient computer handiness). Parents would provide information about hearing and vision of the participant *via* an online parental questionnaire, rather than collecting this “objectively” in-person. The psychophysical tasks would now be collected directly *via* an experiment builder (i.e., Lab.js; Henninger et al., 2020). This provided the quality of stimulus presentation that had a close resemblance to in-person testing. The disadvantage of working directly in the experiment builder was that parents had to download the results and email it to the researcher. The experiment builder allowed us to have consistent and pre-recorded instructions and pre-assembled stimuli that assured the simultaneous presentation of audio and video (as discussed in 3.4). Since it was not possible to control the exact output level of the stimuli on the participants computer, we provided an opportunity for participants to set their individual computer to a level that was comfortable and kept consistent throughout the tasks. The main aim of this study was to see whether children this age showed AV enhancement. This was determined by subtracting participants’ overall percentage correct score of the audio-only trials (i.e., speech in noise combined with a still image) from the percentage correct score from all AV trials (i.e., speech in noise combined with a matching video of the woman speaking). Therefore, results were interpreted as relative levels (i.e., difference in percentages), rather than absolute hearing thresholds. The behavioral tasks, that is, vocabulary (EVT) and phonological awareness (PPA), were assessed using similar methods to in-person testing, over Zoom. Raw scores were used, rather than normed standardized scores due to the limited knowledge of norm interpretation in an online setting (as discussed in 3.7.2). Attention control (catch trials^1^ and random answer options in the 4AFC task) were built-in. Although this is always important when working with children, we focused a bit more on the importance of attentional control online. We note that there are very little data about attentional behavior for online tasks with children (as discussed in 3.6).

### A Symbolic Annotation of Vowel Sounds for Emerging Readers

Although there is an extensive market for educational technologies for literacy (Guernsey and Levine, 2015; Donnelly et al., 2020b), the vast majority of these technologies lack an evidenced-based component (Guernsey and Levine, 2015; Christ et al., 2018) and show small effect sizes (Cheung and Slavin, 2011). It is too often assumed that new implemented technologies will simply be successful. Meta-analyses reveal, however, limited short-and long-term gains, small sample sizes, and less rigorous designs (Blok et al., 2002; Stetter and Hughes, 2010; Grant et al., 2012). Despite this too often assumed “digital magic,” we can specify how technology advantages emerging readers by examining its many opportunities for practice, feedback, motivation, and autonomy in the learning process (Soe et al., 2000; Richardson and Lyytinen, 2014; Wolf et al., 2014; Ronimus and Lyytinen, 2015; Benton et al., 2018; McTigue et al., 2020). And more interestingly, technology provides a platform to supplement more classical learning with individualized materials that struggling readers require, both inside and outside of the classroom. This leads to empowering shared experiences with caregivers.

**Figure 3:**
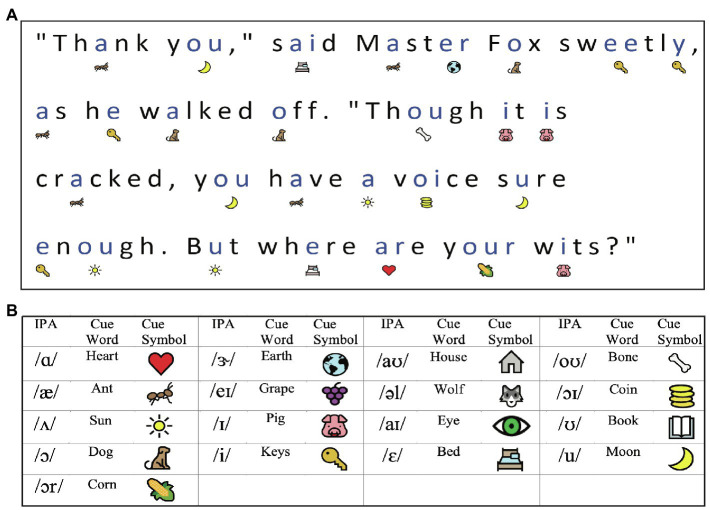
Visualization of the Reading Study*: Sound it Out* provides visual cues under each word that prompts readers on the pronunciation of the vowels contained in the words. Panel (A) presents a sample of a fable passage with symbolic annotations. Panel (B) shows the legend of the image cues used. (Donnelly et al., 2020b).

This study investigated the efficacy of an educational technology to support literacy in 8-to 13-year-old struggling readers (*N*=78), as characterized by performance on a battery of reading assessments. The technology used was specifically designed to scaffold and empower emerging readers (at home and in school). *Sound it Out* is a web-based educational application focusing on phonological awareness and letter-sound correspondence skill. It utilizes visual cues to vowel identity that are placed under the words to scaffold grapheme-phoneme correspondence during connected text reading and was studied in a randomized controlled trial design. As seen in Figure 3, the tool provides visual cues for all vowels in a given text: for example, under the “ou” in “you,” the image of a moon is provided to cue the sound /u/ in /mun/. The aims of the study were to determine whether extended practice with visual cues could produce measurable gains in reading skill. More specifically (1) Can a digital annotation inspired by evidence-based reading practice help children decode novel words, and (2) can this tool help children read more fluently? Lastly, we were interested whether (3) children’s gains were impacted by supervised practice with a caregiver. The study began in the laboratory, with participants asked to attend three in-person appointments for assessment/training with two two-week practice periods at home in between. With the onset of the COVID-19 pandemic, the study was moved online, and this affected study logistics as well as data collection and training fidelity.

The digital literacy app studied was aimed at supporting phonological decoding for both isolated word reading and connected text fluency. In the laboratory research setting, instruction for both child participants and caregivers occurred in-person, with shared attention to teaching materials and a blend of digital/hardcopy materials to maximize learning. Moreover, assessment involved the use of a standard device (tablet) that reduced variability and controlled for potential issues of screen size, resolution, font size, and Internet connectivity. During the first session, all participants (3 groups) completed baseline tests in an uncued condition (without the *Sound it Out* tool). The two intervention groups would then receive training on the app (for more detail see Donnelly et al., 2020a), one group with active caregiver involvement, and one without. These groups would do instructed at home training and come back to the laboratory for a retest session (session 2), by using the cued condition of the app. A refresher training was provided, and another 2weeks with training were repeated to end in a final session 3. The control group completed an identical trajectory, without the cues in the app and without caregiver involvement. We collected five outcome measures at all three time points: decoding accuracy, real-word decoding, pseudo-word decoding, passage reading accuracy, and passage reading rate.

By moving to a virtual setting, the methodology was amended with impacts to the training program, the approach to assessment, and investments in device distribution. Where we could provide the same tablet for all participants in the laboratory, we now offered children the use of their own tablet if preferred. Additionally, all tests were presented digitally, where they were on paper for the in-person version. This added some extra measures to ensure digital consistency, visual presentation of reading passages, and test materials. These adjustments extended the online visits, with a prolonged start to ensure adequate assessment. Another time-intensive aspect was moving the training instructions online. Where initially, the child (and parent) would share a view of the tablet with the researcher who guided them through the app, visually and verbally, they now had to be guided verbally *via* a second screen (a computer) with videoconferencing. Training instruction could be provided at an equal level (as discussed in 3.7.3), but it was definitely more time-intensive.

## Methods; Developing Remote-Friendly Measures for Moderated, Developmental Studies

In order to align with remote research modalities, critical adjustments were made to each study (See Appendix, Table A). For example, all three studies required changes to their respective informed consent procedures and operating logistics. Moreover, in individual task procedures, adjustments were made to presentation mode, video/audio recording format, behavioral measures, and attention maintenance.

### Informed Consent and Privacy

To ensure participants’ understanding, in-person consent procedures are commonly guided by researchers, providing time and space to emphasize or clarify information on the informed consent, such as affirming the participant’s right to withdraw from the study at any time, as well as to address questions and concerns from participants. Comparable procedures can be carried out in virtual studies. Video conferencing platforms (e.g., Zoom, Microsoft Teams, Google Meet) have brought well-appreciated convenience in enabling researchers to moderate consent procedures and online tasks. However, certain privacy and security issues have also been exposed amid the soaring popularity of these platforms. While such issues are heavily dependent upon the individual software’s safety protocol, much responsibility in protecting research subjects lies within institutions and researchers. In our three studies, the research appointments were conducted over Zoom, and for online security purposes, we generated and assigned passcodes and an online waiting room, and to start off the appointments, we reviewed our video/audio protocol with the participant’s caregiver to ensure comprehension of informed consent.

In the Imitation and Reading studies, the majority of the caregivers signed the consent forms prior to the behavioral assessments. For those who were unable to, time was allocated at the beginning of the sessions to address questions and complete consent procedures. In the AV study, parental consent and child assent were both collected *via* audio recording. During all three studies’ consent procedures, no identifiable information was collected. Instead, the research teams generated unique aliases (e.g., multi-digit numeric codes, code names, login credentials) for parents to input for anonymous identification. Links were established between the aliases and participant identities, which were only stored on local computers. Additionally, in the AV and Reading studies (where participants were mature enough to understand study procedures and provide meaningful assent), verbal assent was acquired *via* video or audio recordings.

### Caregiver Involvement

For child studies in laboratory settings, caregiver involvement is often minimized. During the in-person pilot phase of the Imitation study, parents of the 4-year-old participants were invited to view the experimental process from an observation room. In the original designs of the AV and Reading studies involving older children, parents would be asked to stay in a neighboring waiting room or sit at a distance in the experimental room while the study is in session. These strategies removed possible confounds related to caregiver involvement during the task and allowed parents of younger children to monitor the task process and to attend to the children’s needs. When moving these studies online, the caregiver was advised to stay with or near the child during the appointments. Additionally, caregiver roles varied by participants’ age. Among younger children, parental physical assistance is often necessitated for task completion. For instance, to enhance participant compliance, it is typically recommended for a toddler to sit on the parent’s lap or beside the parent in front of the computer, whereas older children tend to have sufficient self-control to perform tasks with less caregiver involvement.

Specifying the role of caregivers in our studies was not only critical to ensuring proper consent, privacy, and children’s comfort, it also helps control parental involvement across families. As such, it was crucial for caregivers to be briefed on research procedures prior to the appointment. In order to uncover the role of caregiver-supervised practice, the Reading study implemented two training/practice conditions: unsupervised, independent reading and supervised, dyadic reading with a caregiver. In the online implementation, this involved providing consistent instructions for caregivers both during laboratory visits and at-home practice sessions. It was also important for caregivers to know what *not* to do. For instance, in a screening task involving picture naming in the Imitation study, parents were allowed to provide hints when children did not recognize an image but were instructed to avoid using word form (i.e., morphological) variations of the targeted word. Parental assistance is also crucial when a research task requires complex manipulation of digital devices. In the AV and Reading studies, participants had to actively interact with a computer or tablet. Although most children at the age of 8–13 (Donnelly et al., 2020b) were able to perform the required manipulations once the app was set up by the caregiver, children aged 6–7 (Gijbels et al., in press) were not equally skillful in manipulating the mouse/trackpad. Therefore, during a training phase, based on participants’ computer proficiency, the researcher made decisions regarding the assistance provided by the parent. If necessary, parents would make mouse clicks, with the limitation that the child had to indicate the answers (by pointing) and the mouse would return to a neutral position in the middle of the screen after every trial.

Typically, parental feedback and parent-guided responses are discouraged in child studies. However, the challenge caused by the unpredictability of caregiver involvement in remote environments can be blunted by deciding prior to the experimental data collection whether parents would assist the child. Because our studies were moderated, researchers could make observations of participants and parents, and as required, instructing parents regarding their participation. In addition to parents receiving instructions at the beginning of each appointment, built-in training phases (as in the Imitation and AV studies) allowed instructions to be repeated to ensure adherence. In addition to the detailed protocols that were verbally communicated to parents prior to the appointments, the research team of the Imitation study also mailed a hardcopy flowchart to help visualize the task procedures. Lastly, because parents are often tempted to help their child “succeed” when they struggle with a task, as the more complex items occur in certain trials, reminder instructions regarding parental intervention were presented throughout the tasks as well.

An additional concern raised with parents is the timing of online appointments. Because they take place in participants’ homes, scheduling has to factor in families’ daily routines and the degree to which it is possible to participate without interruptions. When scheduling virtual appointments, our research teams recommended parents to consider potential distractions throughout a given day and highlighted the importance of creating a quiet environment. We also encouraged parents to schedule appointments when a second caregiver is available to attend to other family members (such as pets and other children), leaving the participant and one parent fully attentive during the appointment. Since home environments are inevitably more distracting (Scott and Schulz, 2017), the research teams prepared parents, prior to the experiment, regarding ways to prevent potential disruptions. In all three studies, we were able to detect and handle interruptions through researcher moderation during the video conference call. However, the challenge for the researcher in these situations is conducting consistent evaluations and accommodations across subjects in order to maintain experimental control (Sheskin and Keil, 2018). We found it critical to establish a set of intervention rubrics beforehand in anticipation of various interruptions and make note of them during the appointments, as well as establishing criteria for data exclusion (e.g., if more than 10% of the trials had to be repeated or if the parent repeatedly violated protocol more than 3 times during a task). For example, in the Imitation study and the phonological awareness and vocabulary component of the AV study, individual stimuli were designed to allow representation when necessary, in order to accommodate sudden “obtrusive interferences” (e.g., significant surrounding noise in the participant’s home, see Appendix, Table B) which were carefully defined prior to the experiment. And such accommodations were marked on the scoring sheet by the researcher.

### Logistical Impacts and Cost of Online Adaptations

Reips (2002) highlighted several logistical advantages of online testing such as increased number of potential participants, lower costs, and accessibility. However, our studies did not benefit significantly in these ways. Recruitment for all three studies used pre-established participant pool databases from the University of Washington. The online procedures reduced participants’ transportation costs (e.g., toll, bus fare, parking) but introduced the cost of mail delivery of equipment and/or testing materials.

Specifically, the Imitation and Reading studies involved providing electronic equipment for participants. To achieve excellent control of audio recordings across participants in the Imitation study, we mailed participants audio recorders, which enabled field recordings of speech production during virtual appointments. Similarly, inherent to the Reading study’s format as a longitudinal experiment with an in-home training component, ensuring access to similar equipment (i.e., touchscreen tablets) was particularly important to the study’s validity.

Both studies benefited from the high level of equipment control. However, equipment handling was a cumbersome process. It required meticulous planning such as schedule forecasting and inventory monitoring. Designated personnel prepared shipments (e.g., instructions/flow charts, equipment, small gifts, return label) sent packages at postal service locations according to the appointment schedules and even personally delivered to families when necessary. Despite the increased workload and logistical complexity caused by transporting research equipment to the families, we accepted this trade-off in order to enhance quality control of data collected in natural environments. Although we acknowledge that this is not feasible for every laboratory, sending equipment gave us the opportunity to reach a population that otherwise would not have access to these studies/ interventions.

A major logistical benefit we encountered across all three studies was increased scheduling and rescheduling flexibility for both researchers and participants. The researchers’ schedule was not subjected to shared laboratory venue availability. Likewise, in addition to work-from-home conditions for many of the parents and school cancelations for children, most families reported increased daytime flexibility. Often, it was easier to squeeze a one-hour virtual appointment into their schedule compared to an in-person visit with commuting and parking difficulties. Similarly, rescheduling appointments and follow-ups with the families were easier compared to previous in-person experiences. Importantly, we could reach families who would have been unable to visit the laboratory (due to distance or availability), which increased the diversity of participants in our studies.

One disadvantage associated with online experiments, as noted by Reips (2002), is a higher attrition rate, which can be addressed by incorporating financial incentives, immediate feedback, and personalization (Frick et al., 2001). We did not notice an increased rate of withdrawal compared to previous in-person studies. We attribute this to study design considerations that were taken in order to provide logistical convenience to the families, as well as financial incentives that were similar to our in-person studies.

### Presentation Mode/Setup

Moving our studies online required substantial adjustments in stimulus presentation and experimental setup. For example, the online Imitation experiment involved cartoon animations that replaced the plush puppets. The end result was visual stimuli that portrayed four cartoon characters whose mouth movements corresponded to pre-recorded audio files. During the online experiment, participants were highly engaged as cartoon characters delivered auditory stimuli. The digital animation showed to be less distracting than the puppet theater setup in the original study design. The online presentation mode eliminated distractions from tangible objects while maintaining a convincing representation of a “talking animal” for children to repeat after and interact with.

In the Imitation and AV studies, cartoon characters narrated task instructions, provided pre-programmed verbal feedback/encouragement, and indicated experimental progress to the participants. For example, the Imitation study provided “food” rewards (e.g., bananas for the monkey character) when children completed a trial, and in the AV study, a star was displayed for every block of trials. These “rewards” served as a progress bar and motivation for the children, and digital presentation offered reliable delivery and consistent timing of the instructions, stimuli, and rewards, which helped reduce unwanted influence from the researcher during facilitation of the tasks.

When presenting auditory stimuli, output levels are important. In laboratory environments, one often uses consistent and calibrated equipment and builds experiments in a virtual environment that provides certain levels of control (e.g., Python). Since there is currently no user-friendly way to run an experiment remotely in virtual environments, the AV study reimagined the experiment by using an online experiment builder. The changes following these adaptations were substantial, but not necessarily noticeable to participants. For example, the AV study required simultaneous presentation of audio and video. We wanted to ensure that potential delays caused by the participant’s computer or browser would not affect the results. Four measures were taken to assure this. First, we pre-compiled the auditory stimuli, the noise files, and the visual part of the stimulus (photo or video). This was done using ffmpeg software (Python 3.7) on the researcher’s computer. Second, these files were then reduced in file size while keeping the quality of the sound and video.^2^ This induced a reduction in loading time. A third precaution taken to assure simultaneous presentation was implementation of a buffer screen (200ms blank screen) before stimulus presentation. This allowed the stimulus to fully load before it needed to be presented. And lastly, we decided to have the participants’ work go directly into the experiment builder (Lab.js), since this would avoid any delays caused by online hosting platforms (e.g., Pavlovia).

Another consideration for remote presentation of auditory stimuli is that exact loudness level on the participants’ end cannot be established. When working at a supra-threshold level, as in these studies, and/or when measuring differences in performance^3^ between auditory stimuli with similar qualities, exact loudness levels are not essential. A similar environment across participants was created by asking participants to set a pre-recorded speech stimulus to a comfortable level and making sure they did not change the audio settings during the experiment.

A third aspect of presenting auditory stimuli is the use of headphones. Although over-ear headphones have been accepted as the gold standard for in-laboratory auditory experiments, for all three online studies, we instructed families to use speakers for all three studies, both to control for audio output variability (compared to using headphones) across devices and to allow easy incorporation of caregiver assistance.

Control of visual presentation is often encouraged. An aspect of this, when designing the experimental setup, is the positioning of the participant, which ideally should be consistent across participants to control for artifacts related to angle, distance, etc. Thus, preset age-and task-specific guidelines could be helpful in remote assessments. In our studies, the participants were asked to sit in a comfortable chair, or on a parent’s lap, with the computer/tablet positioned on a table in front of them. The Imitation and AV studies asked, when possible, to choose a computer over a tablet and to control the size of the display to a certain degree. With these instructions, we expected the camera angle to remain steady throughout the appointments. The Reading study also had a prescient need to ensure that the presentation of text was appropriate and consistent for each study visit. Participants were tested using a tablet (either owned or provided) for study sessions in addition to practice. In doing so, we could control for font size and scroll speed that would be adversely impacted with use of a small screen (i.e., smartphone). In the case of technical glitches that prevented use of the tablets, stimuli were projected onto the participants’ computer screen with considerations made to ensure clear and legible text and visual cues.

### Video/Audio Recording

Where a researcher would be sitting adjacent or opposed to the child in the in-laboratory version of all three experiments, a similar situation was created by administering these tasks *via* a video conferencing tool. Additional to the experimenter’s role, this allowed notes to be taken, questions to be answered, and technical difficulties to be addressed. The flexibility of recording options of these video conferencing tools even facilitated some aspects of our studies.

#### Video Camera Setup

In our studies, Zoom video conference allowed researcher-participant communication, with the stimuli and the participant visible on screen. Similar to the in-person procedures, the Imitation experiment was video-and audio-recorded. The original setup of the study had separate cameras capture the child’s face as well as the puppet show from the child’s perspective. With the online setup, the video conferencing tool offered the convenience of being able to record both angles in the same screen share view field. In the AV experiment, disabling the researcher’s camera allowed the researcher to “hide” as an observer in the background and “appear” during necessary intervention.

#### Audio Setup

Considering the type of measurement (i.e., formant frequencies) in the Imitation study, obtaining quality audio recording is critical to signal analysis. However, Zoom audio recordings are subjected to input setting variability and participants’ choice of microphone. These software and hardware differences can result in incomparable speech signals or missing data. Therefore, in the absence of a highly controlled recording environment and a balanced-input microphone with exacting recording settings (as available in a laboratory booth), we sent each family a small, child-safe^4^ LENA recorder, wearable inside a LENA vest pocket for in-home audio recording during the Zoom appointment. This setup helped minimize the distraction associated with the presence of microphones/recorders and established a controlled distance between the child’s mouth and the recorder. Equipped with a power switch, a record/pause button, and a simple visual feedback mechanism, the recorder was intuitive for families to operate, lowering the risk of user error such as file deletion and data loss. Additionally, all recordings were accessible only through LENA proprietary software on a researcher’s computer. This helped protect participants’ data security especially since the recorders had to be returned to the researcher by mail. Despite the substantive changes introduced in our logistical procedures, sending recording equipment to the participants greatly enhanced the quality of speech data collected, bearing in mind factors that are difficult to control for in-home environments.

Moreover, we acknowledge certain benefits of auditory recordings *via* video conferencing tools of online sessions as was noted in the AV and Reading studies. Occasionally, word productions were not well perceived due to Internet lags and given that this is important for tasks like “speed reading,” one could not ask the participant to repeat the stimulus. However, these “glitches” were mostly absent in audio recordings, and therefore, the test could still be scored reliably.

#### Other Considerations

For some studies, the format (in-person or remote) does not significantly change the implementation of audio/video recording, but recordings can be more efficient when using remote conferencing tools. In the Reading study, in-person sessions required the placement of a recording device (i.e., a handheld audio recorder) near the participant during reading activities. Not only did this introduce variability of recording quality, but perception of an explicit device tends to introduce more “performing” anxiety for child participants. On the contrary, however, we found that parents often reported that recording over the video conferencing platform helped relieve children’s self-consciousness because of the use of a more integrated recording device. For the at-home training sessions, there was no recording, but it was important to log participant adherence to the practice protocol. To achieve this, we implemented online quizzes *via* Microsoft Forms. This provided a simple, secure method for participants to access the quizzes as well as for the research team to track progress.

### Motivation and Sustained Attention

As described by Betts et al. (2006), sustained attention and task load have a big impact on test results for children until the age of 11–12. As children mature, their performance in accuracy and reaction times improves. We conclude that for assessments involving young children, it is important to build in attention control (e.g., catch trials), provide multiple breaks, and decrease task load, especially online.

#### Task Engagement

All three studies focused on designing experiments attractive to children. The Imitation and AV studies were narrated by engaging cartoon characters that served throughout the tasks and/or used in catch trials to stimulate attention. As confirmed by Rhodes et al. (2020), animations are successful in keeping children entertained during experiments. Both children and caregivers provided feedback that these adaptations made the experiments motivating. The Reading study motivated children by choosing reading passages from a variety of topics of interest to children. But more importantly, for struggling readers, a persistent challenge is creating aids that are instructive and fun, given how taxing and frustrating reading is for this demographic. The tool *Sound it Out* was designed using evidence-based practice for reading instruction, but with an element of digital whimsy to help readers decode challenging words.

#### Participant Motivation

In addition to having engaging study designs, motivation can be increased by paying/rewarding subjects (Nussenbaum et al., 2020), as oftentimes, human subject payments are lower for online studies. A financial reward that is communicated to the participant before the start of the experiment or bonus rewards earned by performance can be motivators to complete longer tasks and maintain attention (Nussenbaum et al., 2020). For the studies described, participants were given an online gift card, not based on performance, with an amount similar to that provided for in-person visits. Additionally, the youngest participants received a prize toy resembling one of the cartoon characters featured in the task.

#### Attention Maintenance

**Figure 4:**
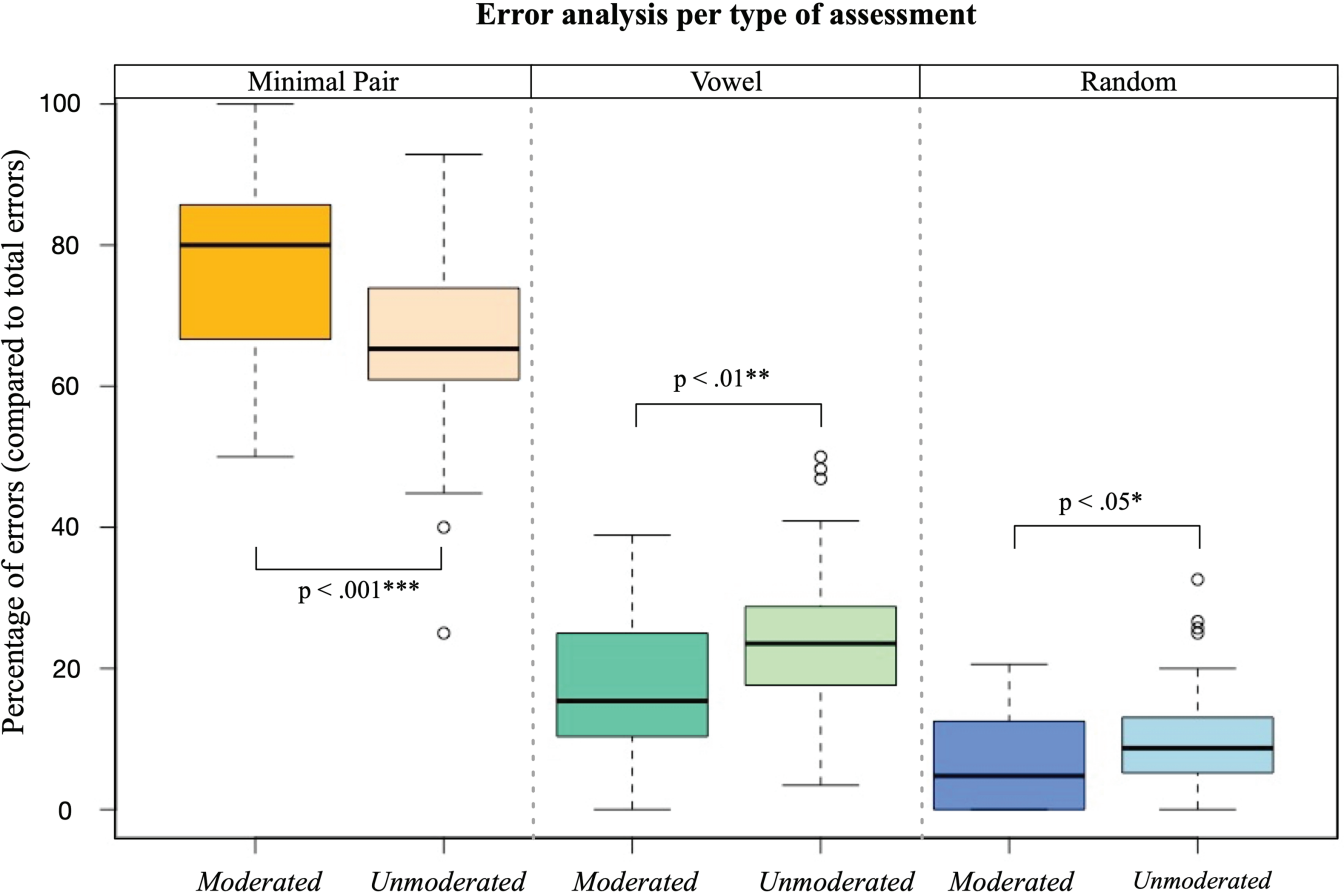
Comparison of a moderated (N=37) and unmoderated (N=47) version of the AV task in 6-to 7-year-olds. The expected error pattern minimal pair > vowel > random errors is shown in both tasks, but more distinct for the moderated task. Random errors, and there for lack of attention is significantly higher in the unmoderated task. Thick horizontal lines represent medians, boxes represent interquartile ranges, and whiskers represent range, excluding outliers. Outliers are defined as values falling more than 1.5 x below or above the 25th and 75th percentiles, respectively, and are shown as circles. Significance: ^*^p<0.05, ^**^ *p*<0.01, ^***^ *p*<0.001.

Attention maintenance is also crucial in child studies. In all three studies, tasks were broken into sections, which allowed children to take breaks. Longer breaks were provided in between tasks. Most children were sufficiently motivated to continue without many breaks, but the opportunities were explicitly offered and even encouraged to those showing waning motivation. Particularly, the AV task had two attention mechanisms built in. First, the cartoon character would appear randomly as catch trials to measure cross-modal attention. Second, general attention was measured by including random answer options. In this 4AFC task, children picked from four answer options. All stimuli were consonant-vowel-consonant words. During the 4AFC presentation, children could pick from the goal stimulus (presented earlier in the audio-only, visual-only or audiovisual modality; e.g., sun), a minimal pair alternative (having one different consonant; e.g., run), an alternative with only the same vowel (e.g., gum), and one with no relationship to the stimulus in meaning or form (e.g., pink). We would not expect children to pick this random answer, unless they did not pay attention to the trial or fail to comprehend task instructions. Because all children were trained to criterion, we believe that random errors could be attributed to a lack of attention. We have facilitated this AV task moderated (*N*=37) and assessed the same task without researcher moderation in a similar group of children (*N*=47, age: 6-to 7-year-olds), as part of a bigger study. As presented in Figure 4, in both moderated and unmoderated assessment, children showed the expected pattern, where most errors were minimal pairs, followed by vowel words and the least responses were random words. We found that this pattern was significantly more distinct for the moderated assessment in every category. Results showed attention in the moderated task was maintained and random errors stayed low throughout the task (*M*=6.63%, SD=7%). A certain level of errors was expected, since we know attention is still developing in this population (Betts et al., 2006). In the unmoderated task, children made significantly more (*t*=−2.26, *p*=0.03*) random errors (*M*=10.14%, SD=7%). This suggests consideration of using moderation or not when developing an online task, depending on the question asked.

### Validity of Online Adaptations

As Whitehead (2007) formulates, in situations where one uses measures online that were initially developed as paper and pencil materials, it is important to demonstrate the equivalence when one wants to interpret these similarly. Confirming existing behavioral norms online for widely used behavioral assessments would greatly benefit this process. More and more studies designed for online testing start to confirm the possibility of getting highly reliable results online in adults (Crump et al., 2013) and children (Sheskin and Keil, 2018), even when the task is pretty different from the initial measure (Yeatman et al., 2021).

Given the nature of virtual assessments, certain factors concerning unequal audio/visual display and environmental differences were beyond our control while facilitating tasks online. However, in order to validate our remote data collection procedures, we were able to establish in-person and online comparisons within several measures critical to each study.

#### Validity Measures in the Imitation Study

**Figure 5:**
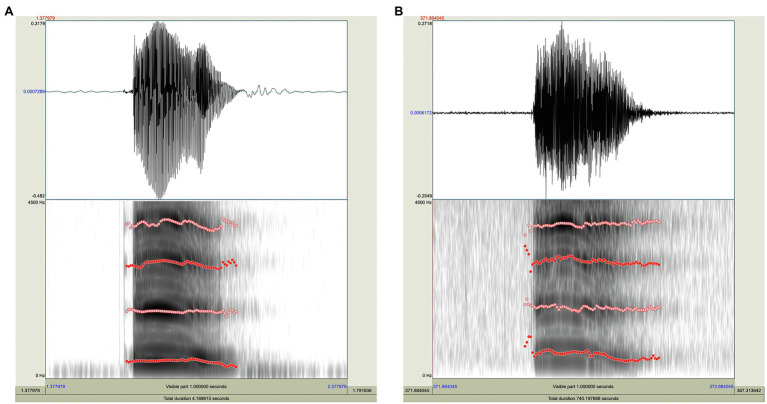
Quality comparison between in-laboratory and in-home audio recording systems in the Imitation study (Cai & Kuhl, in prep). Panel **(A)** shows the spectrogram (with formant tracking) of vowel /ø/, recorded with the laboratory audio recording system, sampled at 44.1kHz. Panel **(B)** shows the spectrogram (with formant tracking) of an in-home LENA recording of the same vowel, sampled at 16kHz.

As mentioned, the collection of speech data in the Imitation study benefited from LENA recorders’ compactness, usability, and security features. However, due to the design rationale behind LENA’s hardware and software systems – intending to capture day-long talk at a time, its recording quality is one 16-bit channel at a 16kHz sample rate (Ford et al., 2008), much lower than the 44.1kHz sample rate common to professional audio recordings for speech analysis. To determine whether LENA recorders were suited for this study, we tested the in-home setup and compared LENA recordings with laboratory audio samples and observed that, despite an expected lower quality in LENA recordings – associated with lower sample rates and higher background noise in natural environments – the vowel formants (i.e., the outcome measures in the study) were equally identifiable in both sets of recordings (see Figure 5).

#### Validity Measures in the Audiovisual Study

Norm-referenced behavioral tasks like vocabulary tasks (e.g., EVT) are extremely valuable in developmental research, especially when researchers are specifically interested in these skills for the target group of participants. This allows the researcher to assure they have a representative group to test their specific hypothesis, and it also allows comparisons with a bigger group of children of the same age or skill level. Since there is currently little information about implementing norm-referenced tests online, a comparison from the AV study of in-person versus moderated online assessment of the EVT is shown below.

Some adaptations needed to be made to move the Expressive Vocabulary task online. Verbal instructions were given (over Zoom) following the assessment manuals, *via* a slideshow instead of the booklet. For some tasks, where children normally would have to point to a picture, the online study required them to verbalize the stimulus or the number/color attached to the picture. For children who could not do this, they were asked to point to the picture on the screen and have the caregiver verbalize it.

**Figure 6:**
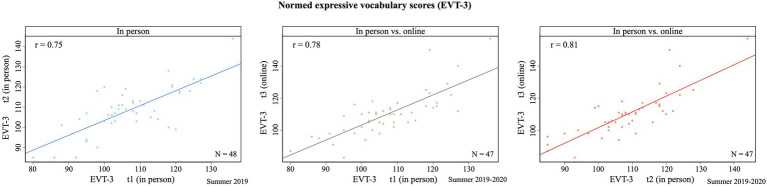
Pearson correlation coefficients between in-person and online testing of a normed expressive vocabulary test (EVT-3), as described in the AV study (N=47; Gijbels et al., in press). There is no significant difference between repeated in-person testing and in-person vs. online testing.

Since there are no data published to date confirming the use of norm-referenced scores for online assessments, we decided to interpret raw scores. This allowed comparing results between children and tasks without overcomplicating data interpretation. Nonetheless, we made a start to validate our results by doing a meta-analysis of in-person and online versions of the same measure. Participants from the online AV study had completed the same vocabulary task (EVT) as part of an in-laboratory study in the summer of 2019. The task was assessed two times (different versions) in-person, with a 3-to 4-week separation. These children did the first version of the test again online in June 2020. The online assessment was facilitated by a trained research assistant and was conducted as similarly as possible to in-person testing. The child, caregiver, and researcher sat in front of their computers with cameras and microphones enabled, and digital scans of the materials were presented in the same way as instructed in the manual, *via* screen sharing. We found a Pearson correlation coefficient of 0.75 between the normed/standard scores of the two in-person assessments. A relationship of 0.78 was between the first (in-person) and third (online) assessment and 0.81 between the second and third assessment (see Figure 6). The correlations between the time points had no statistically significant difference (*p*>0.05), indicating that moderated online assessment of a standardized test like this expressive vocabulary test can be reliable.

#### Validity Measures in the Reading Study

As previously discussed, a primary concern when the Reading study moved to a remote implementation was the ability of a virtual training program for *Sound it Out* to provide comparable benefits to those observed in an earlier, proof-of-concept study (Donnelly et al., 2020a). To our knowledge, the efficacy of remote literacy has not been explored; however, previous work in early childhood language development has shown a significant advantage of in-person learning (Kuhl et al., 2003). Moreover, a recent survey of U.S. teachers observed that most teachers did not feel they were able to deliver the same quality of instruction when using online platforms in response to emergency school closures (Ladendorf et al., 2021).

**Figure 7:**
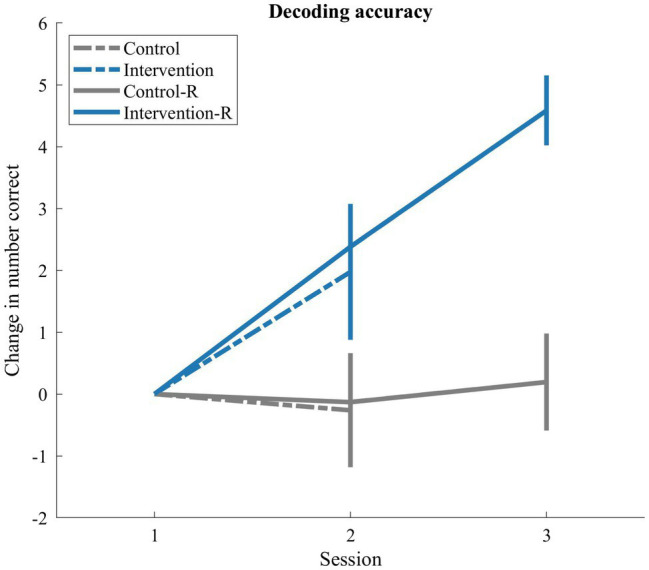
Comparing rate of change for in-person versus remote study. Line plots depict mean change at the group-level for the intervention groups (blue) and control groups (gray) on a composite of real-word and pseudo-word decoding performance. Lines are shown for both a previous, in-person implementation (dotted) and the remote (solid) version delivered in response to the pandemic. Error bars represent +/− 1 SEM.

Contrary to this concern, the Reading study demonstrated comparable-to-enhanced response in comparison with the previous, in-person iteration. As depicted in Figure 7, the rates of change observed after the first, two-week period of training (session 2) for the remote study (solid lines) are parallel to a similar two-week period in the previous study. Data at this shared time point indicate no significant difference between implementations for both control*, t*(42)=−0.02, *p*=0.99, and intervention participant, *t*(69)=−0.63, *p*=0.53, groups. Although future research is needed to determine validity, these data suggest the significant potential for remote literacy training in the context of early childhood research.

### Surprises

As much as we anticipated and prepared for obstacles associated with remote testing (e.g., instructing families to charge or connect their devices to power, conducting A/V testing at the beginning of appointments), occasional issues surfaced in the studies. For example, instead of the recommended device types, one family from the Imitation study used a Kindle tablet and needed to troubleshoot sound settings throughout the appointment due to unstable audio projection. Seldom, but present in all three studies, researchers encountered incidents where participants were disconnected mid-session either due to connection instability or low battery levels.

Overall, adopting the recorder-in-vest setup (see Section “Audio Setup”) resulted in reliable formant analysis in the Imitation study. However, because a few of the participants were not in compliance with wearing the vest, parents had to hold the recorder near the child. In these rare cases, we noticed a few instances of clipping, which is a distortion to an auditory signal when it exceeds the sensor’s constraints on the measurable range of data. In other words, the recorder could have been too close to the child’s mouth, resulting in speech input being too loud for the device.

Additionally, auditory filters and signal-to-noise adjustments on Zoom introduced additional confounds to speech tasks. For example, in the Imitation study, LENA recorders helped the researcher discover rare incidents where caregivers violated our guidelines for caregiver involvement and assisted the child during the imitation task by whispering the sounds. Such knowledge is crucial for data analysis. However, this is often undetectable over Zoom due to its background noise suppression feature. Additionally, auditory misperceptions were observed. For example, a very few participants produced /hi/ when /i/ stimuli were presented to them. Such misperception was not present in our in-person pilot work, and we suspect this to be caused by variability among audio devices and sound settings across participants. We note that the rare instances of misperception occurred only in trials containing the stimulus /i/, and vowel productions in a /h/−onset context have been shown to be virtually identical to those observed in isolation (Kiefte and Nearey, 2017). As a future step, we will explore the option of using experiment builders (as in the AV study) to deliver the stimuli for better control over the variability in audio signal transmissions.

Another data collection-related surprise occurred in the AV study. Visual stimuli included both videos and images. Because these types of stimuli were among our measures of interest, we did not draw attention to them during instruction. Occasional feedback was received about online presentations “not working” because the video seemed to have frozen. We believe this was caused by the caregivers’ realization that technical issues such as choppy videos can occur with studies online, and we suspect participants would question these occurrences less in the laboratory.

In general, we observed that children were more comfortable working from home. Although we initially thought this would lead to more distractions, participants were often less distracted by their familiar home environment than by the “new” laboratory surroundings as experienced in previous studies or pilot phases. Furthermore, it was nice to share this “from home” experience with children we had been working with before in the laboratory – for example, children loved to show their new toys or pets, which created a positive and comfortable environment for the experiments.

## Discussion

In this section, we will first suggest some guiding principles derived from our implementations of the three online studies in order to aid developmental scientists seeking to carry out future online studies. Next, we will look deeper into the current limitations of online behavioral testing involving children as well as some resources and future improvements needed to move the field forward online.

### Guiding Principles Generated From the Three Studies

The studies discussed in this paper differed in research questions explored and age groups involved. However, commonalities and differences among the studies lend themselves to suggesting the following guiding principles for future online developmental studies.

In general, remote consent procedures can take place over secure online portals. But the downside of solely obtaining (electronic) signatures online is the lack of explicit opportunity for participants/caregivers to raise questions and/or concerns. We recognize that it is important to consider consent acquisition as a process rather than a product (Whitehead, 2007), especially when children are involved. Therefore, we posit that a valuable step to take is to ensure participants’ and caregivers’ understanding of informed consent through researcher moderation. This can serve to supplement written consent procedures or can occur as a separately documented process to replace text-based consent forms.

The degree of caregiver involvement is typically determined by the age group and the complexity of equipment manipulation. Involving caregivers of younger participants in our studies required intentional efforts to ensure that they followed the research protocol closely to avoid introducing unwanted interference. Clear communication of research protocols prior to the appointment is crucial in establishing desired caregiver involvement. Additionally, we experienced that it was helpful to provide families visualizations of experimental procedures or scripts of approved caregiver encouragements. Therefore, in addition to a carefully designed protocol, we believe that these steps could help minimize the confounding risk of caregiver interference. Although the level of caregiver involvement differed by age, technical support was critical for all three studies. When active manipulation of technical devices (e.g., mouse clicks) is required by the children, it can be helpful to objectively assess technical proficiency of the child during a training session, and based on the outcome, decisions can be made regarding caregivers’ assistance in technical manipulations.

During data acquisition, it is crucial to generate and deliver consistent stimuli across subjects. However, in remote studies containing visual and auditory stimuli, it is more complicated to ensure this. Each of the three studies attempted to control for the quality of stimuli delivery in their own way, from screen sharing pre-recorded sets of cartoon animations, to providing participants with designated software. Generating and delivering testing materials using experiment builders would be a favorable option as the automation of stimulus delivery has been reported to reduce the workload of the researcher during the task, lowering the chance of human error (Rhodes et al., 2020).

Related to this, we encourage future studies to carefully evaluate the benefits and costs of providing research equipment to the participants following targeted research questions and data types. In our studies, we made logistical decisions based on task designs and resources available. In the Imitation study, mailing LENA recorders and vests to all participating families was a sensible and effective choice because consistency of speech recordings across participants was critical to the experiment. And it was to our unique logistical advantage that we could use existing resources (i.e., the LENA recorders) which happened to be participant-friendly, since the families had participated in our previous research using the same device. Since the Reading study required the use of a tablet-based app, there was a need to mail a tablet to participants who had no access to one. The goal of the study was to provide an intervention/aid for a population that needs help with literacy development. When only including families that own a tablet, a large portion of this population would have been excluded. For the AV study, it was not necessary to send equipment due to the type of data measured. This study took a different approach in experimental control where, through the use of an experimenter builder, general cross-subject consistency in participants’ visual and auditory perception was achieved.

Another helpful measure to ensure experimental control for online developmental studies is researcher moderation. Although most online behavioral procedures can be automated, it is beneficial to control for unexpected changes in the environment, allowing for impromptu adjustments and extra technical support. We suggest from the findings in the AV studies that moderation could help improve participants’ attention. The researcher can be aware of any decline in participants’ attention and suggest a break or introduce adequate motivators. Additionally, researcher moderation allowed participants and their caregivers to ask questions during the consent procedure and ensured that no data would be lost due to invalid consent/assent procedures. Finally, we believe that the personal connection we established with the participants through moderation was beneficial to lowering the attrition rate and helped sustain participants’ attention.

Last but not least, due to the variability and complexity of study designs in developmental research, validation of online methods in this field often stays specific to each study. We believe a potential solution may be to carry out a study design both in-person and remotely during the initial pilot phase and assess the validity of the online study design by comparing pilot results. Moreover, when designing an online study or converting an in-person study to virtual environments, it is consequential to identify areas of adaptation and define the purpose of each adaptation. Meticulous deliberation and systematic documentation of such decisions would maximize the comparability between data collected in-person and remotely and could benefit future replications of the study within or between laboratories.

### Toward a Future of Remote, Moderated Studies of Early Childhood Development

#### Generalizability/Reliability

Researchers desire highly controlled study designs and environments for accurate experimental measures, sometimes at the cost of results generalizability. Virtual settings promote a natural environmental variability, which could increase ecological validity and generalizability (Laugwitz, 2001; Reips, 2002). Depending on the type of research, exploring previously documented findings in naturalistic settings can be useful. Of course, this varies by types of research. As Reips (2002) suggests, behavioral research that is conducted on topics with no relation to computer-mediated communication might make interpretation more selective instead of more generalizable.

With regard to reproducibility of research findings, noise in measurement and contextual factors may compromise reproducibility (Frank et al., 2017). Online methods could make it easier to share digital stimuli, and participants’ environmental control would be comparable from study to study. As online research tasks need to be more automated, participants do not heavily depend on researchers’ involvement in stimulus delivery, reducing interactive bias (Rhodes et al., 2020).

Although Krantz and Dalal (2000) claim equal external validity between in-person and remote testing, currently the comparison between the validity of data collected in-person vs. online is incomplete and needs further evidence. Kim et al. (2019) concluded that, depending on the measure of interest, data collected in-person and online can be comparable or equivalent. They found that replicating in-person studies online did not have a noticeable impact on participants’ response accuracy but affected their reaction time. Reips (2002) added that individual hardware differences, Internet connection, and background running programs can have an effect on data collection consistency across participants and that validity and reliability of online experiments will need to be expanded in the future.

#### Inclusive, Equitable Research

It has been reported that most in-laboratory developmental studies recruit children from areas surrounding universities (Henrich et al., 2010). While online recruitment opens doors to broaden participant recruitment and diversify the subject pool, the diversity is not guaranteed and the change will not happen overnight. Future work is needed to identify barriers to reach diverse populations. According to the National Center for Education Statistics, in 2016, over 80% of the households in the United States have access to the Internet, and in 2018, 90% of the U.S. population owned a desktop computer, laptop, or tablet. This number is increasing every year. Although the numbers with access to technology are high and increasing, there still exist barriers and inequities for online research in a large group of the population, which is associated with lack of access to these resources among certain populations (Neuman and Celano, 2006; Jenkins, 2009). As these are often families of lower income, lower education levels or minorities, online research may bias toward recruiting specific groups of the population, similar to in-person research. Furthermore, research might not be inviting to these hard-to-reach populations. Shaghaghi et al. (2011) point out that it is only possible to reach a wider population if you make active social, cultural, or behavioral adjustments to make the research more meaningful and accessible.

#### Resources

Converting studies online can seem intimidating for many because of the adjustments that need to be made. However, the changes can be quite positive. At times, crises can force adaptation and encourage advancements. Even beyond the pandemic, we believe that online developmental research can be as valuable or even more valuable than in-person research when thoughtful adjustments and considerations are made.

Although we initially felt there was little support for online adaptations from the developmental science literature, we discovered platforms such as Lab.js, Gorilla, and Pavlovia, as well as task forces such as “The Acoustical Society of America’s Task Force on Remote Testing,” which are investing immensely in support systems for researchers interested in virtual studies. Furthermore, other researchers running into similar difficulties while developing online behavioral experiments are starting to report their experiences (e.g.; Sauter et al., 2020). We are hopeful that this trend will continue, and as a result, future studies moving online will benefit from access to more developed systems to start collecting online data with confidence.

## Conclusion

Similar to diverse laboratory-based experimental designs, online methodologies are specific to individual research questions. The three studies mentioned in this paper employed different methods and encountered problems unique to their study design. We hope our experiences will be informative for future remote studies beyond the impact of the COVID-19 pandemic.

We believe by adjusting our developmental research methods from traditional in-person settings to an online format and by acknowledging all the changes needed to be made, our developmental work is as valuable as it would have been in-person. All children could participate from a familiar environment at a time that worked for both them and the researcher, without having to make concessions and, for example, arrive at the laboratory after a long day of school, activities, and driving. Testing from home can positively impact general attention and comfort for children. In our observations, many of our participants wanted to share their world (e.g., toys, pets) with the researcher and were highly motivated to participate. Data collection procedures felt more natural and comfortable for them because they completed the tasks in their home environment. Additionally, we recognize that part of the reason for the ease of our recruitment and the high compliance from our participants could be that we had established strong rapport with most of the participants and their caregivers from previous studies.

All experimental control that would be routine in a laboratory environment had to be reevaluated and adjusted for online testing, which led to carefully considered and documented protocols. This, in combination with the automation of the research tasks, may make it easier for others to replicate our analyses and findings. As our observations (*via* moderation) and results show consistency over participants and home environments, we believe we succeeded in tackling what we initially observed as the most challenging parts of remote developmental work. This goes from finding platforms and technical support to move the experiment online, to control of the participant’s environment and even logistical issues. However, by no means does this paper attempt to license one “correct” set of rules all online developmental research studies should follow. Instead, by sharing our experiences, we would like to call attention to the need for reported evidence of adapted remote studies in this field. We believe that more experiences of online developmental studies remain to be had and shared.

## Data Availability Statement

The raw data supporting the conclusions of this article will be made available by the authors, without undue reservation.

## Ethics Statement

The studies involving human participants were reviewed and approved by University of Washington Human Subjects Division. Written informed consent to participate in this study was provided by the participants’ legal guardian/next of kin. Written informed consent was obtained from the individual(s), and minor(s)’ legal guardian/next of kin, for the publication of any potentially identifiable images or data included in this article.

## Author Contributions

LG, RC, PMD, and PKK contributed to conception and design of the studies. LG, RC, and PMD executed and analyzed the studies. LG and RC wrote the first draft of the manuscript. LG, RC, and PMD wrote sections of the manuscript. All authors contributed to manuscript revision, read, and approved the submitted version.

## Funding

This work was funded by NSF BCS 1551330, NIH NICHD R01HD09586101, NICHD R21HD092771, Microsoft Research Grants and a Jacobs Foundation Research Fellowship to Jason D. Yeatman and by the Overdeck Family Foundation, the University of Washington Institute for Learning & Brain Sciences Ready Mind Project. The funders were not involved in the study design, collection, analysis, interpretation of data, the writing of this article or the decision to submit it for publication.

## Conflict of Interest

The authors declare that the research was conducted in the absence of any commercial or financial relationships that could be construed as a potential conflict of interest.

## Publisher’s Note

All claims expressed in this article are solely those of the authors and do not necessarily represent those of their affiliated organizations, or those of the publisher, the editors and the reviewers. Any product that may be evaluated in this article, or claim that may be made by its manufacturer, is not guaranteed or endorsed by the publisher.

## Footnotes

^1^The catch trials were created by showing a presentation of the cartoon character on top of the stimulus video or image. The children had to yell the cartoon’s name, and this was noted by researchers and parents

^2^ https://handbrake.fr/

^3^Measuring difference of percentage correct performance between AV and audio-only stimuli presentations, rather than absolute thresholds

^4^LENA recorders are child safe, meeting the United States and international safety standards for electronics and toys (see www.lena.org/faqs)

## Appendix

**Table A** Summary description of the three discussed studies. The table provides more detail about the area of expertise, the equipment used, and explains per study degree of moderation, informed consent, caregiver involvement, logistical impact, presentation mode, video and audio recording, motivation and sustained attention, data interpretation, and surprises.

**Table B** Example protocol for handling various types of obtrusive interferences during online facilitation of the Imitation Study. The table categorizes potential interferences that may disrupt data collection and the measures taken (both before and during virtual appointments) to address the disruptions.

## References

[ref1] Abrahamsson N. Hyltenstam K. (2008). The robustness of aptitude effects in near-native second language acquisition. Stud. Second. Lang. Acquis. 30, 481–509. doi: 10.1017/S027226310808073X

[ref2] Benton L. Vasalou A. Berkling K. Barendregt W. Mavrikis M. (2018). A critical examination of feedback in early reading games. *Proceedings of the 2018 CHI Conference on Human Factors in Computing Systems*, 1–12.

[ref3] Betts J. McKay J. Maruff P. Anderson V. (2006). The development of sustained attention in children: the effect of age and task load. Child. neuropsychol. j. normal. abnormal develop. childhood. adolescence. 12, 205–221. doi: 10.1080/09297040500488522 16837396

[ref4] Blok H. Oostdam R. Otter M. E. Overmaat M. (2002). Computer-assisted instruction in support of beginning reading instruction: a review. Rev. Educ. Res. 72, 101–130. doi: 10.3102/00346543072001101

[ref300] Bohner G. Danner U. N. Siebler F. Samson G. B. (2002). Rape myth acceptance and judgments of vulnerability to sexual assault: an Internet experiment. Experimental psychology. 49, 257–269. doi: 10.1026/1618-3169.49.4.257 12455332

[ref600] Cheung A. C. K. Slavin R. E. (2011). The effectiveness of education technology for enhancing reading achievement: a meta-analysis. Best. Evidence. Encyclopaedia. 97, 1–48. PMID: 28764432

[ref6] Christ T. Poonam A. Yu L. (2018). Technology integration in literacy lessons: challenges and successes. Literacy. Res. Instruc. 58, 1–18. doi: 10.1080/19388071.2018.1554732

[ref7] Christiner M. Reiterer S. M. (2013). Song and speech: examining the link between singing talent and speech imitation ability. Front. Psychol. 4:874. doi: 10.3389/fpsyg.2013.00874, PMID: 24319438 PMC3837232

[ref700] Crump M. J. McDonnell J. V. Gureckis T. M. (2013). Evaluating Amazon’s Mechanical Turk as a tool for experimental behavioral research. PloS one. 8:186. doi: 10.1371/journal.pone.0057410 PMC3596391 23516406

[ref8] Donnelly P. M. Gijbels L. Larson K. Matskewich T. Linnerud P. Kuhl P. K. . (2020b). A symbolic annotation of vowel sounds for emerging readers. PsyArXiv. doi: 10.31234/osf.io/akjdr

[ref9] Donnelly P. M. K. Larson T. M. Yeatman J. D. (2020a). Annotating digital text with phonemic cues to support decoding in struggling readers. PLoS One 15:e0243435. doi: 10.1371/journal.pone.0243435 33284838 PMC7721157

[ref10] Duffy M. E. (2002). Methodological issues in web-based research. J. nursing. Scholarship. 34, 83–88. doi: 10.1111/j.1547-5069.2002.00083.x 11901974

[ref800] Ford M. Baer C. Xu D. Yapnel U. Gray S. (2008). The LENA language environment analysis system: audio specifications of the DLP-012 (Technical Report LTR-03-2). Boulder, CO: LENA Foundation.

[ref11] Fort M. Spinelli E. Savariaux C. Kandel S. (2012). Audiovisual vowel monitoring and the word superiority effect in children. Int. J. Behav. Dev. 36, 457–467. doi: 10.1177/0165025412447752

[ref12] Frank M. C. Bergelson E. Bergmann C. Cristia A. Floccia C. Gervain J. . (2017). A collaborative approach to infant research: promoting reproducibility, best practices, and theory building. Infancy. official j. Int. Soc. Infant. Stud. 22, 421–435. doi: 10.1111/infa.12182, PMID: 31772509 PMC6879177

[ref13] Franken M. K. Hagoort P. Acheson D. J. (2015). Modulations of the auditory M100 in an imitation task. Brain. Language. 142, 18–23. doi: 10.1016/j.bandl.2015.01.001 25656319

[ref14] Frick A. Bächtiger M. Reips U. (2001). “Financial incentives, personal information and drop out in online studies,” in Dimensions of Internet Science. eds. Reips U.-D. Bosnjak M. (Lengerich: Pabst), 209–219.

[ref15] Ghazi-Saidi L. Ansaldo A. I. (2017). Second language word learning through repetition and imitation: functional networks as a function of learning phase and language distance. Front. Hum. Neurosci. 11:463. doi: 10.3389/fnhum.2017.00463, PMID: 29033804 PMC5625023

[ref16] Gibson F. Twycross A. (2008). Getting it right for children and young people’s health care services. J. Clin. Nurs. 17, 3081–3082. doi: 10.1111/j.1365-2702.2008.02644.x, PMID: 19012777

[ref17] Gijbels L. Yeatman J. D. Lalonde K. Lee A. K. (in press). Audiovisual speech processing in relationship to phonological and vocabulary skills in first graders. J. Speech Lang. Hear. Res. doi: 10.1177/0265659018793697 PMC9150669 34735292

[ref18] Grant K. W. Bernstein J. G. W. (2019). “Toward a model of auditory-visual speech intelligibility,” in Multisensory Processes (Cham: Springer International Publishing), 33–57.

[ref19] Grant A. Wood E. Gottardo A. Evans M. A. Phillips L. Savage R. (2012). Assessing the content and quality of commercially available reading software programs: do they have the fundamental structures to promote the development of early reading skills in children? NHSA Dialog 15, 319–342. doi: 10.1080/15240754.2012.725487

[ref20] Guernsey L. Levine M. H. (2015). Tap, Click, Read: Growing Readers in a World of Screens. San Francisco: Jossey-Bass.

[ref21] Gweon H. Sheskin M. Chuey A. Merrick M. (2020). Video-chat studies for online developmental research: Options and best practices. *Social Learning Lab Webinar*. Available at: http://sll.stanford.edu/docs/Webinar_materials_v2.pdf (Accessed May 30, 2021).

[ref22] Henninger F. Shevchenko Y. Mertens U. K. Kieslich P. J. Hilbig B. E. (2020). Lab.Js: a free, open, online study builder. Behav. Res. Methods, 1–18. doi: 10.5281/zenodo.597045 PMC9046347 34322854

[ref900] Henrich J. Heine S. J. Norenzayan A. (2010). The weirdest people in the world?. The Behavioral and brain sciences. 33, 61–135. doi: 10.1017/S0140525X0999152X 20550733

[ref23] Hewson C. M. Laurent D. Vogel C. M. (1996). Proper methodologies for psychological and sociological studies conducted via the internet. Behav. Res. Methods. Instrum. Comput. 28, 186–191. doi: 10.3758/BF03204763

[ref24] Holt R. F. Kirk K. I. Hay-McCutcheon M. (2011). Assessing multimodal spoken word-in-sentence recognition in children with normal hearing and children with Cochlear implants. J. Speech Lang. Hear. Res. 54, 632–657. doi: 10.1044/1092-4388(2010/09-0148), PMID: 20689028 PMC3056932

[ref25] Hu X. Ackermann H. Martin J. A. Erb M. Winkler S. Reiterer S. M. (2013). Language aptitude for pronunciation in advanced second language (L2) learners: Behavioral predictors and neural substrates. Brain Lang. 127, 366–376. doi: 10.1016/j.bandl.2012.11.006, PMID: 23273501

[ref26] Jenkins H. (2009). Confronting the Challenges of Participatory Culture: Media Education for the 21st Century. Cambridge, MA: The MIT Press.

[ref27] Jerger S. Damian M. F. Spence M. J. Tye-Murray N. Abdi H. (2009). Developmental shifts in children’s sensitivity to visual speech: a new multimodal picture–word task. J. Exp. Child Psychol. 102, 40–59. doi: 10.1016/j.jecp.2008.08.002, PMID: 18829049 PMC2612128

[ref400] Jerger S. Damian M. F. Tye-Murray N. Abdi A. K. (2014). Children use visual speech to compensate for non-intact auditory speech. J Exp Child Psychol. 126, 295–312. doi: 10.1016/j.jecp.2014.05.003 24974346 PMC4106987

[ref28] Kiefte M. Nearey T. M. (2017). Modeling consonant-context effects in a large database of spontaneous speech recordings. J. Acoust. Soc. Am. 142:434. doi: 10.1121/1.4991022, PMID: 28764432

[ref29] Kim J. Gabriel U. Gygax P. (2019). Testing the effectiveness of the internet-based instrument PsyToolkit: a comparison between web-based (PsyToolkit) and lab-based (E-prime 3.0) measurements of response choice and response time in a complex psycholinguistic task. PLoS One 14:e0221802. doi: 10.1371/journal.pone.0221802, PMID: 31483826 PMC6726137

[ref30] Krantz J. H. Dalal R. (2000). “Validity of web-based psychological research,” in Psychological Experiments on the Internet. ed. Birnbaum M. H. (San Diego, CA: Academic Press), 35–60.

[ref31] Kraut R. Olson J. Banaji M. Bruckman A. Cohen J. Couper M. (2004). Psychological research online. Am. Psychol. 59, 105–117. doi: 10.1037/0003-066X.59.2.105, PMID: 14992637

[ref32] Kuhl P. K. Meltzoff A. N. (1984). The intermodal representation of speech in infants. Infant Behav. Dev. 7, 361–381. doi: 10.1016/S0163-6383(84)80050-8

[ref33] Kuhl P. K. Tsao F. M. Liu H. M. (2003). Foreign-language experience in infancy: effects of short-term exposure and social interaction on phonetic learning. Proc. Natl. Acad. Sci. 100, 9096–9101. doi: 10.1073/pnas.1532872100 12861072 PMC166444

[ref34] Ladendorf K. Muehsler H. Xie Y. Hinderliter H. (2021). Teacher perspectives of self-efficacy and remote learning due to the emergency school closings of 2020. Educ. Media Int., 1–21. doi: 10.1080/09523987.2021.1930481

[ref35] Lalonde K. McCreery R. W. (2020). Audiovisual enhancement of speech perception in noise by school-age children who are hard of hearing. Ear Hear. 41, 705–719. doi: 10.1097/AUD.0000000000000830, PMID: 32032226 PMC7822589

[ref36] Lalonde K. Werner L. A. (2021). Development of the mechanisms underlying audiovisual speech perception benefit. Brain Sci. 11:49. doi: 10.3390/brainsci11010049, PMID: 33466253 PMC7824772

[ref37] Lambert V. Glacken M. (2011). Engaging with children in research. Nurs. Ethics 18, 781–801. doi: 10.1177/0969733011401122, PMID: 21646321

[ref38] Laugwitz B. (2001). “A web experiment on color harmony principles applied to computer user interface design,” in Dimensions of Internet Science. eds. Reips U.-D. Bosnjak M. (Lengerich, Germany: Pabst Science), 131–145.

[ref39] McTigue E. M. Solheim O. J. Zimmer W. K. Uppstad P. H. (2020). Critically reviewing GraphoGame across the world: recommendations and cautions for research and implementation of computer-assisted instruction for word-reading acquisition. Read. Res. Q. 55, 45–73. doi: 10.1002/rrq.256

[ref40] Neuman S. B. Celano D. (2006). The knowledge gap: implications of leveling the playing field for low-income and middle-income children. Read. Res. Q. 41, 176–201. doi: 10.1598/RRQ.41.2.2

[ref41] Nussenbaum K. Scheuplein M. Phaneuf C. Evans M. Hartley C. A. (2020). Moving developmental research online: comparing in-lab and web-based studies of model-based reinforcement learning. Collabra. Psychology. 6. doi: 10.1525/collabra.17213

[ref42] Reips U. (2001). The web experimental psychology lab: five years of data collection on the internet. Behav. Res. Methods. Ins. Comp. 33, 201–211. doi: 10.3758/BF03195366, PMID: 11447673

[ref43] Reips U. (2002). Standards for internet-based experimenting. Exp. Psychol. 49, 243–256. doi: 10.1026/1618-3169.49.4.243, PMID: 12455331

[ref44] Rhodes M. Rizzo M. T. Foster-Hanson E. Moty K. Leshin R. A. Wang M. . (2020). Advancing developmental science via unmoderated remote research with children. J. Cogn. Dev. 21, 477–493. doi: 10.1080/15248372.2020.1797751, PMID: 32982602 PMC7513948

[ref45] Richardson U. Lyytinen H. (2014). The GraphoGame method: the theoretical and methodological background of the technology-enhanced learning environment for learning to read. Hum. Technol. 10, 39–60. doi: 10.17011/ht/urn.201405281859

[ref46] Ronimus M. Lyytinen H. (2015). Is school a better environment than home for digital game-based learning? The case of GraphoGame. Hum. Technol. 11, 123–147. doi: 10.17011/ht/urn.201511113637

[ref47] Ross L. A. Molholm S. Blanco D. Gomez-Ramirez M. Saint-Amour D. Foxe J. J. (2011). The development of multisensory speech perception continues into the late childhood years. Eur. J. Neurosci. 33, 2329–2337. doi: 10.1111/j.1460-9568.2011.07685.x, PMID: 21615556 PMC3127459

[ref48] Sauter M. Draschkow D. Mack W. (2020). Building, hosting and recruiting: a brief introduction to running Behavioral experiments online. Brain Sci. 10:251. doi: 10.3390/brainsci10040251, PMID: 32344671 PMC7226161

[ref49] Scott K. Chu J. Schulz L. (2017). Lookit (part 2): assessing the viability of online developmental research, results from three case studies. Open mind 1, 15–29. doi: 10.1162/OPMI_a_00002

[ref50] Scott K. Schulz L. (2017). Lookit (part 1): a new online platform for developmental research. Open Mind 1, 4–14. doi: 10.1162/OPMI_a_00002

[ref51] Shaghaghi A. Bhopal R. S. Sheikh A. (2011). Approaches to recruiting 'hard-to-reach' populations into research: a review of the literature. Health Promot. Perspect. 1, 86–94. doi: 10.5681/hpp.2011.009, PMID: 24688904 PMC3963617

[ref52] Sheskin M. Keil F. (2018). A video chat platform for developmental research. *TheChildLab.com*. Available at: 10.31234/osf.io/rn7w5 (Accessed June 22, 2021).

[ref53] Sheskin M. Scott K. Mills C. M. Bergelson E. Bonawitz E. Spelke E. S. . (2020). Online developmental science to foster innovation, access, and impact. Trends Cogn. Sci. 24, 675–678. doi: 10.1016/j.tics.2020.06.004, PMID: 32624386 PMC7331515

[ref54] Soe K. Stan K. Juvenna M. C. (2000). Effect of Computer-Assisted Instruction (CAI) on Reading Achievement: a Meta-Analysis. Pacific Resources for Education and Learning. Available at: https://eric-ed-gov.offcampus.lib.washington.edu/?id=ED443079 (Accessed June 19, 2021).

[ref55] Stetter M. E. Hughes M. T. (2010). Computer-assisted instruction to enhance the reading comprehension of struggling readers: a review of the literature. J. Spec. Educ. Technol. 25, 1–16. doi: 10.1177/016264341002500401

[ref56] Whitehead L. C. (2007). Methodological and ethical issues in internet-mediated research in the field of health: an integrated review of the literature. Soc. Sci. Med. 65, 782–791. doi: 10.1016/j.socscimed.2007.03.005 17512105

[ref500] Williams K. (2014). Phonological and Print Awareness Scale. Torrance, CA: WPS Publishing.

[ref57] Williams K. T. (2019). Expressive Vocabulary Test [Measurement instrument]. 3rd *Edn*. Bloomington, MN: NCS Pearson.

[ref58] Wolf M. Gottwald S. Galyean T. Morris R. Breazeal C. (2014). “The reading brain, global literacy and the eradication of poverty,” in Bread and Brain, Education and Poverty. eds. Battro A. Potrykus I. Sorondo M. S. (Vatican City: Pontifical Academy of Sciences), 1–22.

[ref59] Yeatman J. D. Tang K. A. Donnelly P. M. Yablonski M. Ramamurthy M. Karipidis I. I. . (2021). Rapid online assessment of reading ability. Sci. Rep. 11:6396. doi: 10.1038/s41598-021-85907-x 33737729 PMC7973435

