# Supplementary material for: Designing Virtual, Moderated Studies of Early Childhood Development
Source: Front Psychol. 2021 Oct 11;12:740290. doi: 10.3389/fpsyg.2021.740290 (PMC8542922; doi:10.3389/fpsyg.2021.740290)
Supplement: Supplementary file 2 [file Table_2.DOCX]

| **Potential interruptions** | **Pre-appointment instructions** | **Mid-appointment responses** |
| --- | --- | --- |
| External noise or visual distractions such as lawn-mowing, leaf-blowing, pet noises, baby (sibling) crying/needing attention | - Choose appointment times that allow privacy and a quiet environment. - Whenever possible, remove pets and other siblings from the appointment space (if there is a second caregiver available). - Establish an appropriate signal system with the parent to inform researcher of disruptions (e.g. raise hand when the parent needs to get the door unexpectedly) | - Pause the task until a quiet and undisturbed environment is achieved - Make notes of interruptions - Repeat a trial if needed |
| Child participant’s mood swings or physical needs | - Ask parents to take the child’s regular nap/meal schedule into account when scheduling (similar to in-person studies) - Communicate with parents that they may need to calm the child during the appointment - Lay out the task instructions from the child’s perspective, encourage parents to communicate with the child ahead of time to achieve understanding | - Immediately pause the stimuli if child cries, screams, or struggles to get out of the seat/parent’s lap - Inform about progress, and encourage participant (with the parent) to continue. e.g., “You’ve helped three animal friends already, just one left!” “The monkey wants to finish this game with you!” - Incentivize participation with parents (e.g., remind the child of the prize toy at the end of the appointment) - Suggest a snack or stretch break - Upon returning to the task, repeat the instructions and the most recent trial - If rescheduling is needed, mark where the disruption happened as well as the reason, in order to anticipate and prepare for similar issues in the next session |
| Technical difficulties | - Send written instructions to parents regarding the Zoom appointment procedures - Try to use computers instead of tablets and smartphones for the Zoom appointment - If using laptops or tablets, fully charge the device, and keep it connected to power source during the appointment - Recommend testing the videoconferencing setup beforehand with the research team if the family is unfamiliar with Zoom and/or uncertain about general internet connection quality - Account for potential technical issues when estimating appointment duration | - Conduct audio/video testing before starting on the tasks - If the videoconference gets disconnected (either due to connection on the researcher’s or the family’s side), note the task progress, and call the parent to troubleshoot. - Reschedule if needed - Upon returning to the task, repeat the instructions and the most recent trial |
| Parental interference | - Prepare parents regarding the types of involvement that is allowed and discouraged, based on different tasks - Mail hardcopy flowcharts (in order to go over the instructions with the parents at time of appointment with visual aids and reminders) | - Verbal reminders for parents between tasks - In cases of parent’s over-involvement such as providing unwarranted clues to the participant, pause the task, clarify and gently correct parental involvement, and reiterate approved types of involvement |
